# Supplementary material for: MetaQTL: a package of new computational methods for the meta-analysis of QTL mapping experiments
Source: BMC Bioinformatics. 2007 Feb 8;8:49. doi: 10.1186/1471-2105-8-49 (PMC1808479; doi:10.1186/1471-2105-8-49)
Supplement: Additional File 7 — MetaQTL Package : jar file and tutorial. This Zip archive contains both the MetaQTL JAR file and the files of the tutorial. [file 1471-2105-8-49-S7.zip › org.inra.metaqtl/doc/index-files/index-12.html]

M-Index


|  |  |  |
| --- | --- | --- |
| **Overview** \| Package \| Class \| Use \| **Tree** \| **Deprecated** \| **Index** \| **Help** | |  |
| **PREV LETTER**   **NEXT LETTER** | **FRAMES**    **NO FRAMES**     **All Classes** |  |


A B C D E F G H I K L M N O P Q R S T U V W X Y Z 

---


## **M**

**m** - Variable in class org.metaqtl.MultiFitResult: The number of parameters **m1** - Variable in class org.metaqtl.Qtl: The indice of the left flanking marker. **m2** - Variable in class org.metaqtl.Qtl: The indice of the right flanking marker. **main(String[])** - Static method in class org.metaqtl.main.A2Xml: **main(String[])** - Static method in class org.metaqtl.main.ConsMap: **main(String[])** - Static method in class org.metaqtl.main.InfoMap: **main(String[])** - Static method in class org.metaqtl.main.MapView: **main(String[])** - Static method in class org.metaqtl.main.MetaDB: **main(String[])** - Static method in class org.metaqtl.main.MMapView: **main(String[])** - Static method in class org.metaqtl.main.MQTLView: **main(String[])** - Static method in class org.metaqtl.main.QTLClust: **main(String[])** - Static method in class org.metaqtl.main.QTLClustInfo: **main(String[])** - Static method in class org.metaqtl.main.QTLModel: **main(String[])** - Static method in class org.metaqtl.main.QTLProj: **main(String[])** - Static method in class org.metaqtl.main.QTLTree: **main(String[])** - Static method in class org.metaqtl.main.Xml2A: **main(String[])** - Static method in class org.thalia.metaqtl.main.MetaCMS: **main(String[])** - Static method in class org.thalia.metaqtl.main.MetaDraw: **main(String[])** - Static method in class org.thalia.metaqtl.main.MetaMapDraw: **main(String[])** - Static method in class org.thalia.metaqtl.main.MetaSimu: **main(String[])** - Static method in class org.thalia.metaqtl.main.MetaSimuStat: **MAKER\_NAME\_VSPACE** - Static variable in class org.metaqtl.graph.MetaGraphPar: **manageMissingData(int)** - Method in class org.metaqtl.MetaQtlData: Manage the missing data. **MapChartFactory** - Class in org.thalia.bio.entity.factory: General comments must be added here. **MapChartFactory()** - Constructor for class org.thalia.bio.entity.factory.MapChartFactory: **MapCluster** - Class in org.metaqtl: **MapCluster()** - Constructor for class org.metaqtl.MapCluster: Creates a new instance of `MapCluster`. **MapDBFactory** - Class in org.metaqtl.factory: A factory to read the list and the properties of a set of genetic maps. **MapDBFactory()** - Constructor for class org.metaqtl.factory.MapDBFactory: **MapMakerFactory** - Class in org.thalia.bio.entity.factory: **MapMakerFactory()** - Constructor for class org.thalia.bio.entity.factory.MapMakerFactory: **mapName** - Variable in class org.metaqtl.Chromosome: The name of the map in which this chromosome is defined. **mapNames** - Variable in class org.metaqtl.MetaChrom: The names of the genetic maps. **mapOrigin** - Variable in class org.metaqtl.Qtl.QTLProj: The name of the map from which this QTL comes. **MAPPING\_CROSS** - Static variable in interface org.metaqtl.IBioGenomeProperties: The key for mapping cross. **MAPPING\_EXPANSION** - Static variable in interface org.metaqtl.IBioGenomeProperties: The key for mapping expansion status. **MAPPING\_FUNCTION** - Static variable in interface org.metaqtl.IBioGenomeProperties: The key for mapping function. **MAPPING\_FUNCTION\_HALDANE** - Static variable in interface org.metaqtl.IBioGenomeProperties: The code for Haldane mapping function. **MAPPING\_FUNCTION\_HALDANE** - Static variable in interface org.metaqtl.IMetaQtlConstants: Integer code for haldane mapping function **MAPPING\_FUNCTION\_KOSAMBI** - Static variable in interface org.metaqtl.IBioGenomeProperties: The code for Kosambi mapping function. **MAPPING\_FUNCTION\_KOSAMBI** - Static variable in interface org.metaqtl.IMetaQtlConstants: Integer code for kosambi mapping function **MAPPING\_FUNCTION\_UNKNOWN** - Static variable in interface org.metaqtl.IMetaQtlConstants: Integer code for not defined mapping function **MAPPING\_UNIT** - Static variable in interface org.metaqtl.IBioGenomeProperties: The mapping unit key. **MAPPING\_UNIT\_CM** - Static variable in interface org.metaqtl.IBioGenomeProperties: The code for centi-Morgan unit. **MAPPING\_UNIT\_CM** - Static variable in interface org.metaqtl.IMetaQtlConstants: Integer code for centi morgan mapping unit **MAPPING\_UNIT\_M** - Static variable in interface org.metaqtl.IBioGenomeProperties: The code for Morgan unit. **MAPPING\_UNIT\_M** - Static variable in interface org.metaqtl.IMetaQtlConstants: Integer code for morgan mapping unit **MAPPING\_UNIT\_UNKNOWN** - Static variable in interface org.metaqtl.IMetaQtlConstants: Integer code for unknown mapping unit **MappingFunction** - Class in org.metaqtl.util: define all cartogarphies functions **MappingFunction()** - Constructor for class org.metaqtl.util.MappingFunction: **MapRawFactory** - Class in org.thalia.bio.entity.factory: **MapRawFactory()** - Constructor for class org.thalia.bio.entity.factory.MapRawFactory: **mapScale** - Variable in class org.metaqtl.Qtl.QTLProj: The scale ratio between the original map and the map on which the QTL has been projected. **MapTabFactory** - Class in org.thalia.bio.entity.factory: **MapTabFactory()** - Constructor for class org.thalia.bio.entity.factory.MapTabFactory: **MapView** - Class in org.metaqtl.main: **MapView()** - Constructor for class org.metaqtl.main.MapView: **mapWeight** - Variable in class org.metaqtl.Qtl.QTLProj: The cumulated scale weights of the common marke sequences between the original map and the map on which the QTL has been projected. **Marker** - Class in org.thalia.bio.entity: Class Description Here **Marker()** - Constructor for class org.thalia.bio.entity.Marker: **Marker(String, IBioEntity)** - Constructor for class org.thalia.bio.entity.Marker: **MARKER** - Static variable in interface org.thalia.bio.IBioLocus: **MARKER\_NAME\_FONT** - Static variable in class org.metaqtl.graph.MetaGraphPar: **MARKER\_POSITION\_FONT** - Static variable in class org.metaqtl.graph.MetaGraphPar: **MARKERLOCUS** - Static variable in interface org.thalia.bio.IBioEntityTypes: **mcidx** - Variable in class org.metaqtl.CMarkerSequence: The common marker indices on the chromosomes. **merge(GeneticMap)** - Method in class org.thalia.bio.entity.GeneticMap: **Deprecated.**  **mergeGenome(IBioGenome, IBioGenome)** - Static method in class org.thalia.bio.util.BioUtilities: This method merges two genomes in a single one : `g1` is modified and will contain the result of the merge. **MetaAlgorithm** - Class in org.metaqtl.algo: **MetaAlgorithm()** - Constructor for class org.metaqtl.algo.MetaAlgorithm: **MetaChrom** - Class in org.metaqtl: A MetaChrom is the result of the merge of the members of a `ChromCluster`. **MetaChrom(MultiFitResult, ChromCluster)** - Constructor for class org.metaqtl.MetaChrom: Get a new instance of `MetaChrom` which is initialized by using the result of the merge of the members of a `ChromCluster`. **MetaCMS** - Class in org.thalia.metaqtl.main: **MetaCMS()** - Constructor for class org.thalia.metaqtl.main.MetaCMS: **MetaDB** - Class in org.metaqtl.main: **MetaDB()** - Constructor for class org.metaqtl.main.MetaDB: **MetaDico** - Class in org.metaqtl: This class represents a dictionnary of synonyms. **MetaDico()** - Constructor for class org.metaqtl.MetaDico: **MetaDicoFactory** - Class in org.metaqtl.factory: **MetaDicoFactory()** - Constructor for class org.metaqtl.factory.MetaDicoFactory: **MetaDraw** - Class in org.thalia.metaqtl.main: **MetaDraw()** - Constructor for class org.thalia.metaqtl.main.MetaDraw: **MetaGraph** - Class in org.metaqtl.graph: **MetaGraph()** - Constructor for class org.metaqtl.graph.MetaGraph: **MetaGraphPar** - Class in org.metaqtl.graph: **MetaGraphParFactory** - Class in org.metaqtl.factory: **MetaGraphParFactory()** - Constructor for class org.metaqtl.factory.MetaGraphParFactory: **MetaMain** - Class in org.metaqtl.main: **MetaMain()** - Constructor for class org.metaqtl.main.MetaMain: **MetaMap** - Class in org.metaqtl: A `MetaMap` is a bundle of `MetaChrom` objects. **MetaMap(int)** - Constructor for class org.metaqtl.MetaMap: Creates a new instance of `MetaMap` which will contain `nchr` `MetaChrom` objects. **MetaMapAdapter** - Class in org.metaqtl.adapter: This class defines some usefull methods to adapt `MetaMap` objects into other kinds of objects or the inverse. **MetaMapAdapter()** - Constructor for class org.metaqtl.adapter.MetaMapAdapter: **MetaMapAlgorithm** - Class in org.metaqtl.algo: This class is dedicated to the construction of a consensus genetic map from a set of genetic maps based on a Weigthed Least Square approach. **MetaMapAlgorithm(IBioGenome[], IBioGenome, IBioLocus[], MetaDico)** - Constructor for class org.metaqtl.algo.MetaMapAlgorithm: **MetaMapDraw** - Class in org.thalia.metaqtl.main: **MetaMapDraw()** - Constructor for class org.thalia.metaqtl.main.MetaMapDraw: **MetaMapSummaryFactory** - Class in org.metaqtl.factory: A factory to write out the information on a `MetaMap`. **MetaMapSummaryFactory()** - Constructor for class org.metaqtl.factory.MetaMapSummaryFactory: **metaQtlAnalysis** - Variable in class org.metaqtl.Chromosome: The results of a meta-analysis of QTL. **MetaQtlAnalysis** - Class in org.metaqtl: A QTL meta analysis consists in a clustering of QTL positions over chromosomes for different group of traits. **MetaQtlAnalysis(int)** - Constructor for class org.metaqtl.MetaQtlAnalysis: Creates a new instance of a `MetaQtlAnalysis` **MetaQtlAnalysisAdapter** - Class in org.metaqtl.adapter: **MetaQtlAnalysisAdapter()** - Constructor for class org.metaqtl.adapter.MetaQtlAnalysisAdapter: **MetaQtlAnalysisFactory** - Class in org.metaqtl.factory: A factory to deal with the result of a QTL meta-analysis. **MetaQtlAnalysisFactory()** - Constructor for class org.metaqtl.factory.MetaQtlAnalysisFactory: **MetaQtlAnalysisModelFactory** - Class in org.metaqtl.factory: **MetaQtlAnalysisModelFactory()** - Constructor for class org.metaqtl.factory.MetaQtlAnalysisModelFactory: **MetaQtlAnalysisSummaryFactory** - Class in org.metaqtl.factory: A factory to deal with QTL meta-analysis result. **MetaQtlAnalysisSummaryFactory()** - Constructor for class org.metaqtl.factory.MetaQtlAnalysisSummaryFactory: **MetaQtlData** - Class in org.metaqtl: QTL meta analysis first requires to gather the QTL according to the group trait they belong and then to format the QTL data into data points to perfom clustering. **MetaQtlData(Qtl[])** - Constructor for class org.metaqtl.MetaQtlData: Create a new instance of `MetaQtlData` which will represent the given array of `Qtl`. **MetaQtlModel** - Class in org.metaqtl: **MetaQtlModel()** - Constructor for class org.metaqtl.MetaQtlModel: **MetaQtlModel(int)** - Constructor for class org.metaqtl.MetaQtlModel: **MetaQtlModelFactory** - Class in org.metaqtl.factory: **MetaQtlModelFactory()** - Constructor for class org.metaqtl.factory.MetaQtlModelFactory: **MetaQtlResult** - Class in org.metaqtl: A `MetaQtlResult` is used to store the results of a QTL meta analysis which has been performed for a given trait group on a given chromosome. **MetaQtlResult()** - Constructor for class org.metaqtl.MetaQtlResult: **MetaQtlResult(String, int)** - Constructor for class org.metaqtl.MetaQtlResult: **MetaSimu** - Class in org.thalia.metaqtl.main: **MetaSimu()** - Constructor for class org.thalia.metaqtl.main.MetaSimu: **MetaSimuStat** - Class in org.thalia.metaqtl.main: **MetaSimuStat()** - Constructor for class org.thalia.metaqtl.main.MetaSimuStat: **METHOD** - Static variable in class org.metaqtl.algo.HClustAlgorithm: A global variable to control the custering method. **mfc** - Variable in class org.metaqtl.Chromosome: The mapping function for this chromosome **mir** - Variable in class org.metaqtl.EMCriteria: **MMapView** - Class in org.metaqtl.main: **MMapView()** - Constructor for class org.metaqtl.main.MMapView: **mode** - Variable in class org.metaqtl.QtlPartition: The mode of representation **models** - Variable in class org.metaqtl.MetaQtlModel: The number of Qtl per chromosome and per trait. **mqtls** - Variable in class org.metaqtl.Chromosome: The meta Qtls on this chromosome. **MQTLView** - Class in org.metaqtl.main: **MQTLView()** - Constructor for class org.metaqtl.main.MQTLView: **mr** - Variable in class org.metaqtl.Chromosome: The adjacent recombination rate between ordered markers **mrkNames** - Variable in class org.metaqtl.Chromosome: The ordered marker names **mrkNames** - Variable in class org.metaqtl.MetaChrom: The ordered marker names. **mrkOc** - Variable in class org.metaqtl.MetaChrom: The number of occurence per marker oved chromosomes. **mStep(double[], double[], EMResult, double)** - Static method in class org.metaqtl.algo.EMAlgorithm: The Maximization step : here this step is straighforward and simple analytical formula are applied to obtain the new parameter estimates. **mu** - Variable in class org.metaqtl.EMResult: The means of the clusters. **MultiFitResult** - Class in org.metaqtl: This class represents a result of a multilinear regression analysis. **MultiFitResult(int, int)** - Constructor for class org.metaqtl.MultiFitResult: Creates a new instance of `MultiFitResult` with the given number of observations `N` and parameters `M`. **mut** - Variable in class org.metaqtl.Chromosome: The mapping unit for this chromosome

---


|  |  |  |
| --- | --- | --- |
| **Overview** \| Package \| Class \| Use \| **Tree** \| **Deprecated** \| **Index** \| **Help** | |  |
| **PREV LETTER**   **NEXT LETTER** | **FRAMES**    **NO FRAMES**     **All Classes** |  |


A B C D E F G H I K L M N O P Q R S T U V W X Y Z 

---
